# A role for organ level dynamics in morphogenesis of the *C. elegans* hermaphrodite distal tip cell

**DOI:** 10.1242/dev.203019

**Published:** 2024-10-09

**Authors:** Theadora Tolkin, Julia Burnett, E. Jane Albert Hubbard

**Affiliations:** Department of Cell Biology, NYU Grossman School of Medicine, New York, NY 10016, USA

**Keywords:** Morphogenesis, *C. elegans*, Niche, Distal tip cell, Germ line

## Abstract

The morphology of cells *in vivo* can arise from a variety of mechanisms. In the *Caenorhabditis elegans* hermaphrodite gonad, the distal tip cell (DTC) elaborates into a complex plexus over a relatively short developmental time period, but the mechanisms underlying this change in cell morphology are not well defined. We correlated the time of DTC elaboration with the L4-to-adult molt, but ruled out a relevant heterochronic pathway as a cue for DTC elaboration. Instead, we found that the timing of gonad elongation and aspects of underlying germline flux influence DTC elaboration. We propose a ‘hitch and tow’ aspect of organ-level dynamics that contributes to cellular morphogenesis, whereby germline flux drags the flexible DTC cell cortex away from its stationary cell body. More broadly, we speculate that this mechanism may contribute to cell shape changes in other contexts with implications for development and disease.

## Teaser

**Summary:** Flux in the underlying germ line contributes to complex morphogenesis of the adult hermaphrodite distal tip cell in *C. elegans*.

## INTRODUCTION

Cell shape is dynamic. Although *in vitro* studies have contributed immensely to our understanding of the intrinsic mechanisms governing cell shape, cell diversity and dynamics *in vivo* suggest that organismal context plays an important role. Here, morphogenesis of the *Caenorhabditis elegans* distal tip cell (DTC), which is the germline stem cell niche, revealed an unexpected role for organ-level dynamics in the generation of a complex cell morphology over developmental time.

A striking feature of the hermaphrodite DTC is its dramatic morphogenesis between larval and adult stages (Byrd et al., 2014; Linden et al., 2017; Wong et al., 2013). In larval stages, the DTC is cap shaped. Between the mid-L4 larval stage and day 1 of adulthood, the DTC forms a large plexus (Byrd et al., 2014), a morphogenetic event termed DTC elaboration (Linden et al., 2017).

In response to the DTC-expressed membrane-bound DSL family ligands LAG-2 and APX-1, distal germ cells activate a Notch family receptor, GLP-1 (see Hubbard and Schedl, 2019 and references therein), the activity of which interferes with meiotic entry, thus maintaining stem cell identity and a proliferative state. Germline proliferation in the blind-ended distal gonad pushes germ cells in the proximal direction, away from the DTC (Rosu and Cohen-Fix, 2020). After loss of GLP-1 activity, germ cells undergo an average of one division before entering prophase of meiosis I (Fox and Schedl, 2015), and eventually forming gametes (first sperm, then oocytes). Oocytes mature and are ovulated into the spermatheca in response to signals from the sperm (Huelgas-Morales and Greenstein, 2018). Thus, the adult hermaphrodite germ line undergoes distal-to-proximal flux, provided sperm are present.

Adult DTC morphology is variable and complex, as noted by previous studies (Byrd et al., 2014; Finger et al., 2003; Fitzgerald and Greenwald, 1995; Gordon et al., 2020, 2019; Henderson et al., 1994; Tolkin et al., 2022). Byrd et al. (2014) described the adult DTC plexus with defined anatomical features and established its dependence on underlying stem cells (Byrd et al., 2014). The adult DTC possesses a variable number of processes running proximally from the cell body along the surface of the gonad, each with a variable number of branches that intercalate between germ cells (Byrd et al., 2014). The number, length and width of processes is non-stereotyped (Byrd et al., 2014), and the exact number of underlying germ cells that contact the DTC and its processes varies between gonads, though such contact is thought to promote GLP-1 signaling (Lee et al., 2016).

The mechanisms driving DTC elaboration are unclear. DTC processes do not extend over differentiated germ cells (Byrd et al., 2014; Linden et al., 2017), nor do they form in the male gonad (Crittenden et al., 2019), suggesting that specific aspects of hermaphrodite gonadogenesis influence DTC elaboration. An RNA interference (RNAi) screen for DTC elaboration defects targeting an initial 708 genes identified 145 candidates, many of which also affect development (Linden et al., 2017). Twenty-nine validated hits included genes with diverse and, in many cases, general cellular functions. The site of action of some of these genes was attributed to the DTC and/or germline, raising the possibility of extensive interdependence (Linden et al., 2017). This interdependence creates an experimental challenge whereby manipulations that impact DTC morphology are difficult to distinguish from indirect effects owing to the effect of the manipulation on the germ line.

We wished to further understand the mechanisms underlying DTC elaboration. Although previous work determined that elaboration occurred within a 24-h window between the L4 and adult (Byrd et al., 2014), we performed a detailed time-course analysis and established a more precise correlation to the time of the L4-to-adult molt. We tested and ruled out the *lin-41* heterochronic pathway as a cue for DTC elaboration. Instead, manipulating the timing of gonad elongation revealed a role for the cessation of gonad elongation. Further, inhibiting germline flux significantly reduced DTC elaboration, suggesting that DTC elaboration partially depends on the movement of germ cells away from the DTC. In addition, increasing DTC membrane deformability led to enhanced and precocious DTC elaboration. Finally, certain aspects of elaboration are affected by loss of E-cadherin and L1CAM adhesion.

Based on these observations, we propose a mechanism whereby germline dynamics contribute to DTC morphology. We propose that an overall reversal of germline flux drags the flexible and adherent DTC cell cortex away from the stationary cell body. More broadly, we speculate that this mechanism – in which potentially signaling-active membranes are towed along by signal-receiving cells – may contribute to cell shape changes in other contexts with implications for development and disease.

## RESULTS

### The beginning of DTC elaboration correlates with the L4-to-adult molt

**Fig. 1.**
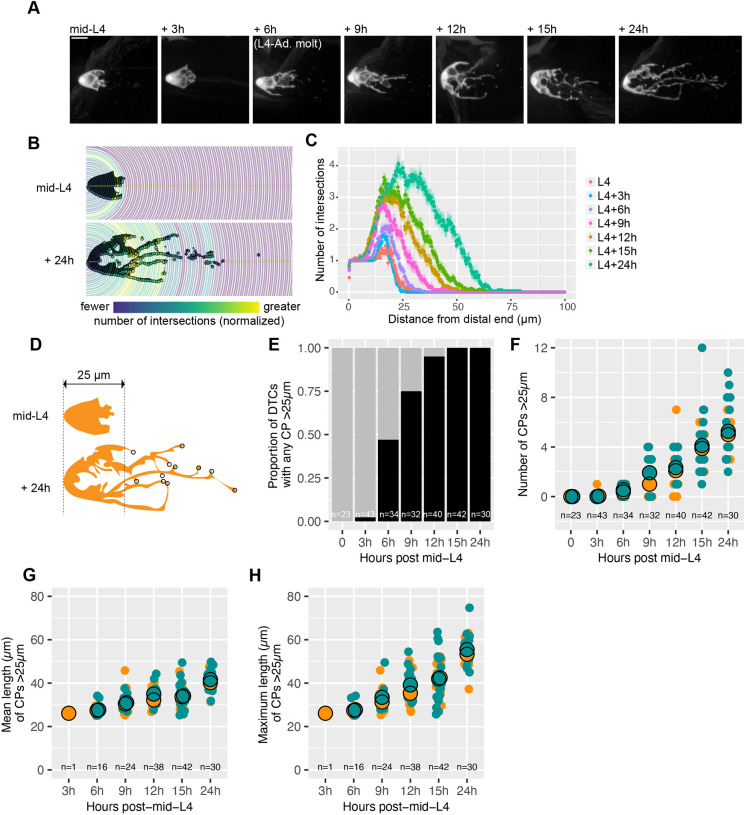
**Quantification of distal tip cell (DTC) morphology in a detailed time course over 24 h post-mid-L4.** (A) Representative live images of DTCs at the indicated time points. Each image is from a different worm. DTC marker is *qIs57(lag-2p::GFP)*. Scale bar: 10 µm. (B) Representation of Sholl radii and corresponding intersection markers in L4 and L4+24 h DTCs. Heat map represents the number of intersections at each radius, normalized to the maximum for that image. (C) Average number of intersections for each Sholl radius across all DTCs for each time point. Each dot with bars represents the mean±s.e.m. (D) Examples of manual DTC analysis in L4 and adult DTCs (same images as in B). Circles indicate end-points of CPs >25 µm. (E) Proportion of all DTCs at each time point with any CPs >25 µm. Black and gray bars represent DTCs with and without CPs >25 µm, respectively. Data pooled from three replicates after multiple pairwise Fisher's exact tests to confirm that pooling was appropriate. (F) Number of CPs >25 µm for each DTC at each time point. (G) Mean length of CPs >25 µm for each DTC with any CPs >25 µm. (H) Maximum length of CPs >25 µm for each DTC with any CPs >25 µm. In all dot plots, colors represent separate biological replicates; small dots represent a single DTC; large dots represent mean for that replicate. The total *n* for all replicates in each condition is indicated.

The *C. elegans* DTC undergoes a striking morphological change between the last larval stage and early adulthood: the cap-like larval DTC grows in size and complexity, characterized by cellular processes extending proximally. The complex and variable architecture of the adult DTC has inspired several analyses of adult DTC morphology (Byrd et al., 2014; Gordon et al., 2020; Linden et al., 2017; Tolkin et al., 2022). These analyses indicated a one-day time window for elaboration. To further resolve the time course, we imaged DTCs at mid-L4 and at time points post-mid-L4: L4 plus 3 h (L4+3 h), L4+6 h, L4+9 h, L4+12 h, L4+15 h and L4+24 h (Fig. 1A; see Materials and Methods).

We used two methods to quantify DTC morphology at each time point, the first being Sholl analysis (Fig. 1B,C). This unbiased method was originally developed for neuronal morphometry (Ferreira et al., 2014; Sholl, 1953). Here, it quantifies the frequency of intersections between DTC features and concentric radii. The method does not distinguish between previously described subtypes of processes such as short intercalating (SIPs) and long extending (LEPs) processes, and fragments (Byrd et al., 2014). The cap-like larval DTCs at L4 and L4+3 h show just one or two intersections, and there were no intersections beyond a radius of about 25 µm at larval time points. Beginning at L4+6 h, which corresponds to the L4-to-adult molt, the number of intersections increases, as does the number of DTCs with any intersections beyond 25 µm, both increasing with successive time points (Fig. 1C).

In addition to automated Sholl analysis, we quantified DTC elaboration using a manual morphometric analysis (Gupta et al., 2024). This analysis defines the length and number of continuous processes (CPs), including branches, by their end-points (Fig. 1D). Based on the Sholl analysis indicating that larval DTCs do not extend beyond 25 µm, we included only those CPs that ended >25 µm away from the distal end of the gonad. We quantified the proportion of DTCs with any CP longer than 25 µm (Fig. 1E), and for each DTC (even those with no CPs longer than 25 µm), we quantified the number of CPs >25 µm (Fig. 1F). For DTCs that had at least one CP >25 µm, we also quantified the average length of CPs >25 µm per DTC and the length of the longest CP (Fig. 1G,H).

Consistent with our Sholl analysis, manual counting showed that the proportion of DTCs with any CPs extending beyond 25 µm from the distal end surpassed 50% only after L4+6 h (Fig. 1E). The number, mean length and maximum length of CPs increased steadily at each subsequent time point (Fig. 1F-H).

In short, both methods provide a similar picture wherein DTC elaboration beyond 25 µm begins in a time frame coincident with the L4-to-adult molt. Our subsequent analyses utilized the manual marking method (Gupta et al., 2024).

### The LIN-41 heterochronic pathway does not influence the timing of DTC elaboration

Because DTC elaboration begins around the L4-to-adult molt, we considered the possibility that the *lin-41* heterochronic pathway might cue DTC elaboration. LIN-41, a TRIM family protein, acts downstream of *let-7* to inhibit adult-specific developmental programs. Reduced *lin-41* activity leads to precocious adult programs, and overexpression leads to a retarded phenotype (Ambros and Horvitz, 1984; Del Rio-Albrechtsen et al., 2006; Slack et al., 2000). If *lin-41* were a cue, we would expect that reducing *lin-41* would cause precocious DTC elaboration, whereas elevating *lin-41* activity would delay its onset. We therefore quantified DTC morphology in *lin-41* RNAi and *lin-41(bx37gf)*.

**Fig. 2.**
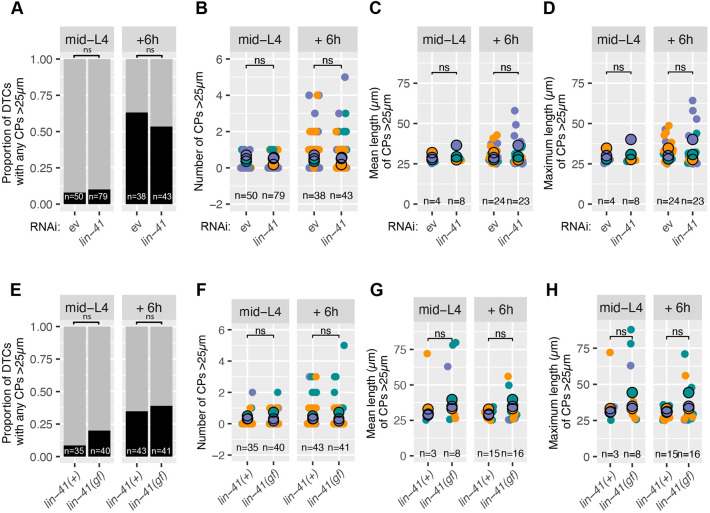
**The *lin-41* heterochronic pathway does not impact DTC elaboration.** (A-D) Data for worms raised on bacteria carrying either empty vector (ev) or a plasmid targeting *lin-41* for RNAi. (A) Proportion of all DTCs with any CPs >25 µm. Data pooled from three replicates after multiple pairwise Fisher's exact tests to confirm that pooling was appropriate. Fisher's exact test: L4 *P*=0.7652; molt *P*=0.499. (B) Number of CPs >25 µm for each DTC. Two-sided Wilcoxon test: L4 *P*=0.69; molt *P*=0.27. (C) Mean length of CPs >25 µm for each DTC with any CPs >25 µm. Two-sided *t*-test: L4 *P*=0.14; molt *P*=0.51. (D) Maximum length of CPs >25 µm for each DTC with any CPs >25 µm. Two-sided *t*-test: L4 *P*=0.14; molt *P*=0.57. (E-H) Wild-type [*lin-41(+)*] or *lin-41(bx37)* [*lin-41(gf)*] worms, as indicated. (E) Proportion of all DTCs with any CPs >25 µm for the genotypes indicated. Data pooled from three replicates after multiple pairwise Fisher's exact tests to confirm that pooling was appropriate. Fisher's exact test: L4 *P*=0.2028; molt *P*=0.8216. (F) Number of CPs >25 µm for each DTC. Two-sided Wilcoxon test: L4 *P*=0.18; molt *P*=0.81. (G) Mean length of CPs >25 µm for each DTC with any CPs >25 µm. Two-sided *t*-test: L4 *P*=0.82; molt *P*=0.2. (H) Maximum length of CPs >25 µm for each DTC with any CPs >25 µm. Two-sided *t*-test: L4 *P*=0.83; molt *P*=0.17. DTC marker is *qIs57(lag-2p::GFP)*. In A and E, black and gray bars represent DTCs with and without CPs >25 µm, respectively. In all dot plots, colors represent separate biological replicates; small dots represent a single DTC; large dots represent mean for that replicate. The total *n* for all replicates in each condition is indicated. ns, not significant.

We quantified DTC morphology at mid-L4 and at L4+6 h (adult molt) in worms subjected to *lin-41* RNAi feeding (Timmons et al., 2001) (see Materials and Methods) beginning in the L1 stage. We found no significant difference between control worms and *lin-41* RNAi-fed worms with respect to all parameters measured (Fig. 2A-D), suggesting that *lin-41* does not provide a cue for DTC elaboration. Similarly, we observed no significant difference between *lin-41(+)* and *lin-41(gf)* worms either at mid-L4, or at the presumptive time of the L4-to-adult transition (L4+6 h), in any parameters measured (Fig. 2E-H).

Therefore, despite the temporal correlation between DTC elaboration and the larval-to-adult transition, we conclude that the LIN-41 heterochronic pathway does not cue DTC elaboration.

### DTC elaboration and the completion of gonad morphogenesis temporally coincide

**Fig. 3.**
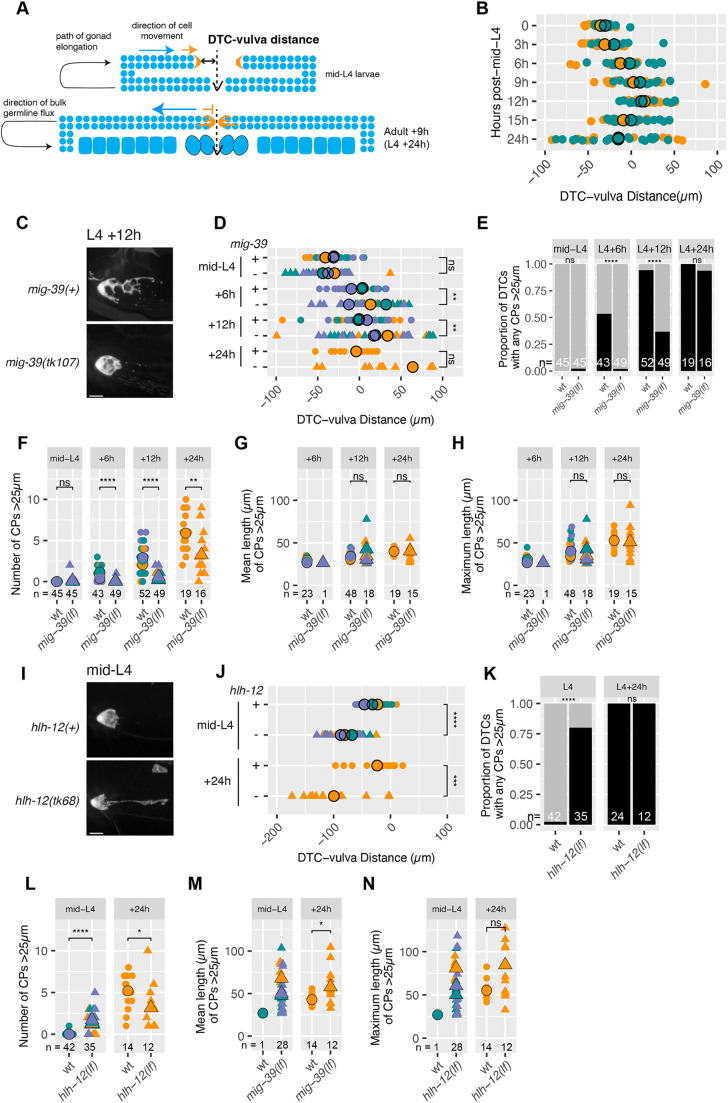
**Gonad elongation cessation correlates with DTC elaboration.** (A) Schematic of gonad elongation. Germ cells are in blue, and the DTC is in orange. Dashed lines indicate the vulval midline. Solid black lines and arrows indicate the path of gonad elongation or the distance between the vulval midline and the distal end of the gonad, where indicated. Blue and orange arrows indicate the direction of movement for germ cells and DTC, respectively. (B) Time course of gonad elongation showing the distance between the vulval midline and the distal end of the gonad. (C) Representative images of DTCs in wild-type (top) and *mig-39(tk107)* (bottom) early adult (L4+12 h) worms. (D) Time course of gonad elongation comparing wild type (circles) and *mig-39(tk107)* (triangles) showing the distance between the vulval midline and the distal end of the gonad. Two-sided *t*-test. (E) Time course comparing the proportion of DTCs with any CPs >25 µm in wild type (wt) and *mig-39(tk107)* [*mig-39(lf)*], as indicated. Data pooled from three replicates after multiple pairwise Fisher's exact tests to confirm that pooling was appropriate. (F) Time course comparing the number of CPs >25 µm in DTCs of wild type (circles) and *mig-39(tk107)* worms (triangles). Two-sided Wilcoxon test. (G) Time course comparing the mean length of CPs >25 µm for each DTC with any CPs >25 µm in wild type (circles) and *mig-39(tk107)* (triangles). Two-sided *t*-test. (H) Time course comparing the maximum length of CPs >25 µm for each DTC with any CPs >25 µm in wild-type (circles) and *mig-39(tk107)* worms (triangles). Two-sided *t*-test. (I) Representative images of DTCs from wild-type (top) and *hlh-12(tk68)* (bottom) worms in early adulthood (L4+12 h). (J) Time course of gonad elongation comparing wild type (circles) and *hlh-12(tk68)* (triangles). Superplot shows the distance between the vulval midline and the distal end of the gonad. Two-sided *t*-test. (K) Time course comparing the proportion of DTCs with any CPs >25 µm in wild type and *hlh-12(tk68)*, as indicated. Fisher's exact test. (L) Time course comparing the number of CPs >25 µm in wild type (circles) and *hlh-12(tk68)* (triangles). Two-sided Wilcoxon test. (M) Time course comparing the mean length of CPs >25 µm for each DTC with any CPs >25 µm in wild type (circles) and *hlh-12(tk68)* (triangles). Two-sided *t*-test. (N) Time course comparing the maximum length of CPs >25 µm for each DTC with any CPs >25 µm in wild type (circles) and *hlh-12(tk68)* (triangles). Two-sided *t*-test. In M and N, because only a single wild-type DTC produced a CP >25 µm at the mid-L4 stage, we could not perform statistical comparisons. In all panels where significance is indicated with asterisks: ****P<0.00001; ***P<0.0001; **P<0.001; *P<0.01; ns, not significant (*P*≥0.01). Scale bars: 10 µm. DTC marker in images is *qIs57(lag-2p::GFP).* In E and K, black and gray bars represent DTCs with and without CPs >25 µm, respectively. In all dot plots, colors represent separate biological replicates; small dots represent a single DTC; large dots represent mean for that replicate. The total *n* for all replicates in each condition is indicated.

Gonad elongation occurs within the same developmental time window as DTC elaboration, so we examined the relationship between the two. Gonad elongation initiates ventrally, then reflexes dorsally in the L3, turning towards and then moving along the dorsal side toward the middle of the worm before terminating around the time of the L4-to-adult molt (Cecchetelli and Cram, 2017; Cram et al., 2006; Imanishi et al., 2020; Kikuchi et al., 2015; Littleford et al., 2021). As a proxy for gonad elongation during the final phase, we measured the distance between the distal end of the gonad arm and the midline axis (Fig. 3A).

Using the same time points as the DTC elaboration time course (see Fig. 1), we observed that between L4+3 h and L4+9 h, the distal end of the gonad moves, on average, towards and then slightly past the midline (Fig. 3B), with less average distance covered per unit time between L4+9 h and L4+12 h. The precise time and position of elongation is variable, so we cannot determine the exact time point of termination using our population-level analysis. Therefore, either the average speed of gonad elongation slows after L4+9 h, or some gonads completely stop elongating between L4+9 h and L4+12 h, while others continue to elongate. Between L4+15 h to L4+24 h, the distal end appeared to regress behind the midline, likely owing to expansion of the uterus and the loop region (Atwell et al., 2015).

In sum, a close correlation exists between slowing or cessation of gonad elongation and DTC elaboration.

### The cessation of gonad elongation influences DTC elaboration

To assess causality between the cessation of gonad elongation and the initiation of DTC elaboration, we examined worms in which cessation of elongation is delayed or premature. Gonad elongation has been well characterized (Agarwal et al., 2022; Cecchetelli and Cram, 2017); gonads can prolong elongation upon loss of the transcription factor *mig-39* (Kikuchi et al., 2015), and, conversely, elongation can terminate early upon loss of the transcription factor *hlh-12* (also known as *mig-24*) (Tamai and Nishiwaki, 2007).

If they are related, we would expect persistent elongation to reduce DTC elaboration and early termination of gonad elongation to cause precocious DTC elaboration. We quantified DTC elaboration in worms carrying the null alleles *mig-39(tk107)* or *hlh-12(tk68)*. We quantified the DTC-vulval distance and DTC elaboration in worms without gross confounding pathfinding defects at mid-L4, L4+6 h, L4+12 h and L4+24 h in *mig-39(tk107)* and *mig-39(+)* (Fig. 3C). As expected, gonads elongated significantly farther in *mig-39(tk107)* (Fig. 3D). At L4+6 h, only 2% of DTCs in *mig-39(tk107)* had CPs longer than 25 µm, versus 53% in the wild type. At L4+12 h the difference was still marked (37% versus 92%). By L4+24 h, although nearly all DTCs in the *mig-39* mutant had at least one CP >25 µm and these CPs attained similar lengths as in the wild type, the number of CPs was significantly lower (averaging three versus six CPs) (Fig. 3E-H) (see Discussion). Thus, abnormally prolonging gonad elongation resulted in delayed CP accumulation.

We then analyzed gonad elongation and DTC morphology in *hlh-12(tk68)* mutant worms compared with wild type in gonads that completed the U-turn [97%, *n*=58 in *hlh-12(+)*; 83%, *n*=60 in *hlh-12(tk68)*]. As expected, in *hlh-12(tk68)* worms, gonad elongation along the dorsal route was severely stunted at mid-L4 and L4+24 h (Fig. 3J). At the L4 stage, the percentage of DTCs with any CPs >25 µm was significantly higher in *hlh-12(tk68)* (80% versus 2.4%) (Fig. 3K). DTCs in *hlh-12(tk68)* worms also produced more numerous and longer CPs (Fig. 3L-N). These results suggest that premature cessation of gonad elongation correlates with precocious DTC elaboration. However, we also note that the morphology of *hlh-12(tk68)* DTCs did not phenocopy normal adult DTC morphology as they often had a single long process (Fig. 3I), suggesting that an additional role for *hlh-12* in DTC elaboration may exist.

We conclude that cessation of gonad elongation strongly influences the timing and extent of DTC elaboration.

### Bulk germline flux promotes DTC elaboration

How might gonad elongation and DTC elaboration be related? One consideration is that cessation of gonad elongation correlates temporally with an overall reversal in the direction of germline flux: in larval stages, the gonad elongates and germ cells, on average, move in the same direction as the DTC. In contrast, once gonad elongation stops, overall germ cell flux reverses and germ cells, on average, move away from the DTC (Fig. 3A). We hypothesized that germline flux may be important for DTC elaboration.

**Fig. 4.**
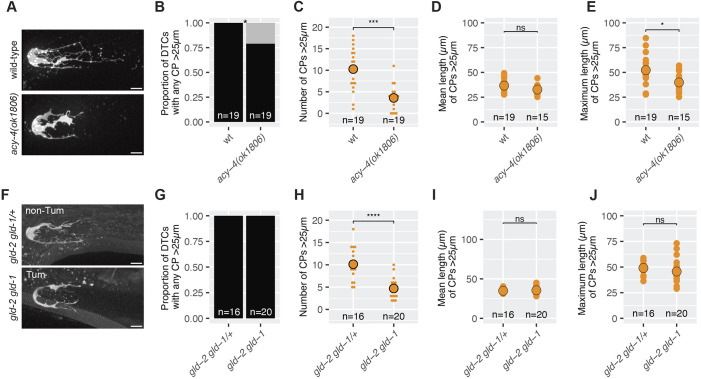
**Bulk germline flux promotes DTC elaboration.** (A-E) Data for DTCs marked with *naIs37(lag-2p::mCherry)* from either wild type (wt) or worms carrying the *acy-4(ok1806)* loss-of-function allele, as indicated. (A) Representative images of L4+24 h DTCs in live worms that were either wild type at the *acy-4* locus (top) or carrying the *acy-4(ok1806)* mutation. (B) Proportion of DTCs with any CPs >25 µm. Fisher's exact test. (C) Number of CPs >25 µm. Two-sided Wilcoxon test. (D) Mean length of CPs >25 µm for each DTC with any CPs >25 µm. Two-sided *t*-test. (E) Maximum length of CPs >25 µm for each DTC with any CPs >25 µm. Two-sided *t*-test. (F-H) Data for worms of the genotype *gld-2(q497) gld-1(q485)/hT2* or their *gld-2(q497) gld-1(q485)* homozygous siblings, as indicated, with DTCs marked by *naSi8(lag-2p::GFP)*. (F) Representative images of Day 1 adult (L4+24 h) DTCs in live worms that were either heterozygous for the *gld-2 gld-1* double mutation (i.e. non-Tum; top) or their homozygous siblings (i.e. Tum; bottom). (G) Proportion of DTCs with any CPs >25 µm. Fisher's exact test. (H) Number of CPs >25 µm in each genotype. Two-sided Wilcoxon test. (I) Mean length of CPs >25 µm for each DTC with any CPs >25 µm. *t*-test: *P*=0.18. (J) Maximum length of CPs >25 µm for each DTC with any CPs >25 µm. *t*-test: *P*=0.26. In all panels where significance is indicated with asterisks: ****P<0.00001; ***P<0.0001; *P<0.01; ns, not significant (*P*≥0.01). Scale bars: 10 µm. In B and G, black and gray bars represent DTCs with and without CPs >25 µm, respectively. In all dot plots, colors represent separate biological replicates; small dots represent a single DTC; large dots represent mean for that replicate. The total *n* for all replicates in each condition is indicated.

One aspect of germline flux is bulk flux proximally due to cytoplasmic flow, oocyte growth and ovulation of oocytes (Nadarajan et al., 2009; Wolke et al., 2007). We examined DTC elaboration in *acy-4(ok1806)* worms, in which oocytes fail to mature. In this mutant, oocytes stack up in the proximal gonad, blocking bulk flux (Govindan et al., 2009). At L4+24 h, *acy-4(ok1806)* worms had a significantly smaller proportion of DTCs with any CPs >25 µm [79% versus 100% in *acy-4(+)*] (Fig. 4B). The number of CPs >25 µm was also significantly fewer (averaging four versus ten CPs) (Fig. 4C). Furthermore, the maximum length of CPs >25 µm was significantly shorter (39 versus 52 µm; Fig. 4E), although the mean length of CPs >25 µm was not (Fig. 4D). Thus, impairing bulk germline flux by blocking ovulation impacts both CP number and length.

Germline tumors, such as in *gld-2 gld-1* double mutants (Kadyk and Kimble, 1998), also impair bulk germline flux. We compared DTC elaboration in tumorous (Tum) *gld-2 gld-1* double-mutant worms with balanced heterozygous, non-tumorous (non-Tum) siblings (Fig. 4F). At L4+24 h, all DTCs had at least one CP >25 µm (Fig. 4G). However, DTCs in Tum worms had significantly fewer CPs than non-Tum (averaging 4.7 compared with ten; Fig. 4H), although the mean and maximum CP lengths were not significantly different (Fig. 4I,J).

These observations support the hypothesis that bulk germline flux contributes to DTC elaboration.

### Distal germ cell movement away from the DTC promotes DTC elaboration

Smaller scale germ cell flux occurs in the distal germ line. Proliferating germ cells in the progenitor zone (PZ) are displaced proximally, away from the distal end of the gonad (see blue and orange arrows in Fig. 3A; Rosu and Cohen-Fix, 2017). We reasoned that germline progenitor cell proliferation that drives germ cells away from the DTC (Crittenden et al., 2006; Maciejowski et al., 2006; Rosu and Cohen-Fix, 2017) may also contribute to DTC elaboration.

One way to interfere with germline progenitor cell proliferation is to reduce GLP-1*/*Notch activity*,* which is required to maintain the proliferative fate. Previous results demonstrated that late larval or adult loss of *glp-1* interferes with DTC elaboration and maintenance of DTC processes. One interpretation of these results is that DTC architecture depends on germ cell fate (Byrd et al., 2014) such that the DTC does not elaborate around meiotic germ cells (Linden et al., 2017).

Another, not mutually exclusive, interpretation is that germline progenitor cell proliferation per se also contributes to DTC elaboration because it supports distal germ cell flux. Using physical and genetic strategies, we sought to interfere with germline progenitor cell proliferation (and presumed proximal germ cell movement) without interfering with the proliferative germ cell fate.

**Fig. 5.**
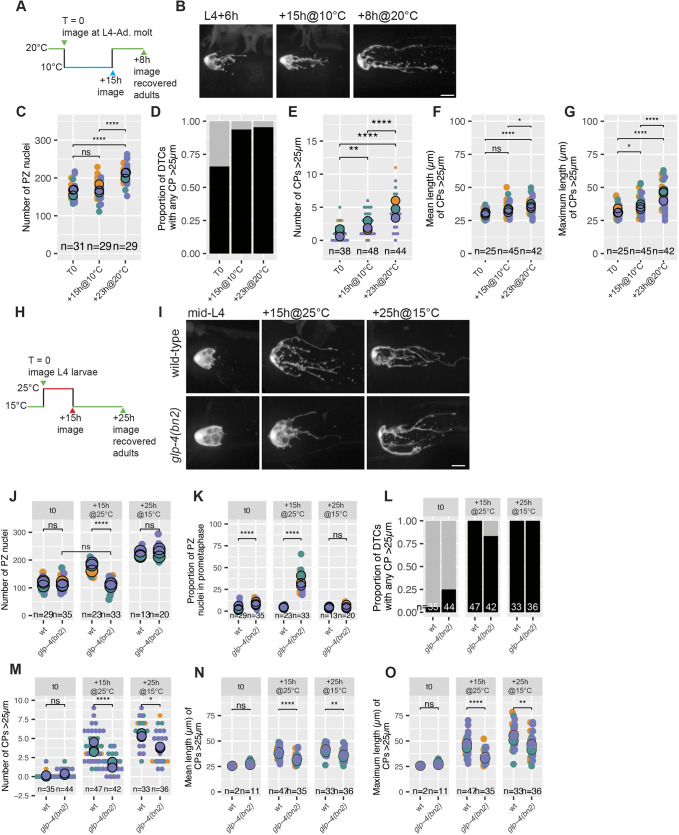
**Distal germ cell cycle progression promotes DTC elaboration.** (A) Schematic of the cold temperature experiment. Worms were raised at 20°C until mid-L4+6 h, when some worms were sampled for DAPI staining or live imaging. The remaining worms were shifted to 10°C for 15 h, after which additional samples were taken. The remainder were returned to 20°C for 23 h, and then analyzed. (B) Representative live images of DTCs at the indicated time points. (C) Number of PZ nuclei at each time point. Two-sided *t*-test. (D) Proportion of DTCs with any CPs >25 µm at each time point. Fisher's exact test. (E) Number of CPs >25 µm for each DTC at each time point. Two-sided Wilcoxon test. (F) Mean length of CPs >25 µm for each DTC with any CPs >25 µm at each time point. Two-sided *t*-test. (G) Maximum length of CPs >25 µm in each DTC with any CPs >25 µm. Two-sided *t*-test. (H) Schematic of *glp-4(bn2)* experiment. Worms were raised at 15°C until the mid-L4 stage and were sampled for DAPI staining or live imaging. The remaining worms were shifted to 25°C for 15 h, after which more samples were taken. The remainder were returned to 15°C for 25 h and analyzed. (I) Representative live images of DTCs of the indicated genotypes at the indicated time points. (J) Number of nuclei in the progenitor zone. Two-tailed *t*-test. (K) Proportion of nuclei in each gonad that were deemed to be in prometaphase by nuclear morphology. Two-sided *t*-test. (L) Proportion of DTCs with any CPs >25 µm at each time point. Fisher's exact test. (M) Number of CPs >25 µm in each DTC. Two-sided Wilcoxon test. (N) Mean length of CPs >25 µm in each DTC with any CPs >25 µm. Two-sided *t*-test. (O) Maximum length of CPs >25 µm in each DTC with any CPs >25 µm. Two-sided *t*-test. In all panels where significance is indicated with asterisks: ****P<0.00001; **P<0.001; *P<0.01; ns, not significant (*P*≥0.01). Scale bars: 10 µm. In all panels, the DTC marker is *qIs57(lag-2p::GFP).* In D and L, black and gray bars represent DTCs with and without CPs >25 µm, respectively. In all dot plots, colors represent separate biological replicates; small dots represent a single DTC; large dots represent mean for that replicate. The total *n* for all replicates in each condition is indicated.

The cell cycle acutely and reversibly arrests at low temperature (Sulston and Horvitz, 1977). To avoid interfering with somatic gonad development, we shifted worms from 20°C to 10°C at the L4-to-adult molt and kept worms at 10°C for 15 h (15h@10°C) (Fig. 5A). At the beginning and end of cold incubation, we quantified the number of PZ cells and DTC morphology in samples of fixed and live worms, respectively. To assess reversibility, we shifted worms back to 20°C for 8 h (15h@10°C+8h@20°C) and again quantified the number of PZ cells and DTC morphology (Fig. 5B-G).

The PZ pool did not significantly increase during the 15 h exposure to low temperature (Fig. 5C), indicating a slowed or arrested cell cycle, although the proportion of DTCs with CPs >25 µm increased (t0: 66%; t+15h@10°C: 94%) (Fig. 5D), as did the average number of CPs >25 µm (t0: 1.2; t+15h@10°C: 2.2) (Fig. 5E). Nevertheless, a comparison with the 20°C time course (Fig. 1F) suggests that the cold shift affected DTC elaboration: by 15 h after the molt (∼21 h post-mid-L4), the expectation would be approximately five CPs >25 µm, rather than the approximately three CPs seen after 15 h at 10°C. In addition, unlike at 20°C, the mean length of CPs >25 µm did not significantly increase at 10°C, and the maximum length of CPs >25 µm increased only slightly, reaching an average of ∼35 µm after 15 h at 10°C (Fig. 5E-G), much shorter than the observed average of ∼50 µm when grown at 20°C (Fig. 1). In addition, bulk flux persisted at low temperature as evidenced by the formation of oocytes in worms kept at 10°C for 15 h after the molt. This persistent bulk flux may account for the partial inhibition of early CP formation after cold temperature exposure. Together, these results suggest that CP extension at/after the molt either relies partially on cold-sensitive mechanisms and/or is influenced by distal germline flux.

We next turned to genetic means to interrupt germline cell cycle progression without causing germ cell differentiation. We assessed *emb-30(tn377)*, a temperature-sensitive allele that blocks metaphase-to-anaphase transition at 25°C (Furuta et al., 2000). Worms were shifted to 25°C at the mid-L4 stage, and we quantified the percentage of PZ nuclei in metaphase after 6, 12 and 24 h. By 12 h, however, the nuclear morphology of distal germ cells was abnormal, precluding further analysis (Fig. S1A). Samples from L4+6 h showed only 20% of nuclei in metaphase, and DTC elaboration was not affected (Fig. S1B-E).

We then assessed a valyl aminoacyl tRNA synthetase, GLP-4 (Rastogi et al., 2015); the *glp-4(bn2)* allele inhibits germline mitotic progression in a reversible, temperature-sensitive manner (Beanan and Strome, 1992). Arrested germ cell nuclei exhibit a dotted appearance, interpreted as prometaphase. To assess the proportion of arrested germ cells, we quantified the number of dotted nuclei within the PZ in *glp-4(bn2)* and control worms raised at 15°C and shifted to the restrictive temperature starting from the mid-L4 (Fig. 5H,I). After 15 h at 25°C, the number of PZ cells in *glp-4(bn2)* worms did not increase, compared with the wild type (Fig. 5J). Moreover, ∼30% of PZ nuclei displayed a morphology consistent with prometaphase, compared with 5% in wild type (Fig. 5K). Upon down-shift to the permissive temperature, *glp-4(bn2)* worms recovered the PZ pool.

This same condition altered DTC morphology in *glp-4(bn2)*. A significantly smaller proportion of DTCs in *glp-4(bn2)* had any CPs >25 µm (83.3% versus 100%) (Fig. 5L), and fewer CPs >25 µm were seen in *glp-4(bn2)* (average 1.5 versus four). Furthermore, the mean and maximum lengths of CPs >25 µm were shorter in *glp-4(bn2)* than in wild type (26 µm versus 38 µm, and 28 µm versus 46 µm, respectively) (Fig. 5M-O).

In summary, the two conditions that robustly interfered with progenitor cell accumulation, cold temperature and *glp-4(bn2)*, impaired DTC elaboration. These results are consistent with a model in which distal germ cell proliferation is one of several factors contributing to DTC elaboration.

### DTC elaboration correlates with distal germline flux in individual gonads

Given the population-level correlation between distal germ cells and DTC elaboration, we assessed their relationship on an individual gonad level. We marked germline nuclei with photoconvertible Dendra fused to histone H3.3 (Gleason et al., 2023), similar to a previous study in adults (Rosu and Cohen-Fix, 2017, 2020).

**Fig. 6.**
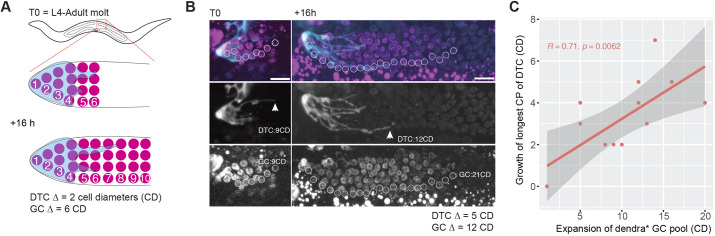
**Distal germ cell flux correlates with DTC process outgrowth in individual worms.** (A) Schematic of the germ cell photoconversion experiment. Marked by H3.3::Dendra2, germ cells underlying the DTC were photoconverted and re-imaged 16 h later. (B) Representative images of a single gonad arm immediately after photoconversion, and at re-imaging 16 h later. White circles surround the ventral-most photoconverted germ cell nucleus at each successive cell diameter distance from the distal end of the gonad. Arrowheads indicate the proximal end of the longest CP. (C) Correlation between the growth of the longest CP and the expansion of the photoconverted germ cell pool. Each dot represents a single DTC. Pearson correlation coefficient R=0.71 and *P*=0.0062. Scale bars: 10 µm.

We photoconverted germline progenitors based on the position of our DTC marker at the L4-to-adult molt and quantified the proximal extent of the germ cell pool and the DTC longest CP [in numbers of germ cell diameters (CDs) from the distal end] (Fig. 6A). After growth in a free-living environment for 16 h, we re-imaged the same gonad arm in the same individual, and performed the same measurements of the proximal reach (in CDs), which included converted germ cells and their descendants that carried the photoconverted protein (Fig. 6A,B). We found a strong correlation (R=0.71) between the expansion of the photoconverted germ cell pool and the increase in the longest DTC process length in individual worms (Fig. 6C). Although we cannot be certain that the longest CP at t0 is the same CP measured as longest at t16, we never observed a DTC process extending beyond the front of photoconverted cells and their progeny, which would have indicated a flux-independent or active mechanism of DTC process growth.

These results are consistent with the possibility that DTC process elongation is advanced by the movement of underlying germ cells away from the DTC.

### NMY-2/non-muscle myosin restricts DTC elaboration

**Fig. 7.**
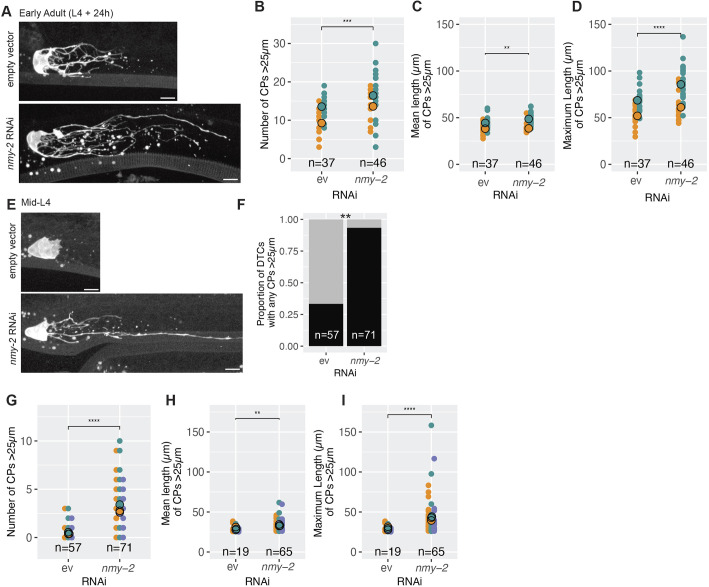
**Loss of *nmy-2* leads to increased size and complexity of the DTC plexus and precocious DTC elaboration.** (A-D) Data for worms at L4+24 h. Representative images of DTCs in control (top) and DTC-specific *nmy-2* RNAi at L4+24 h. (B) Number of CPs >25 µm in each DTC. Two-sided Wilcoxon test. (C) Mean length of CPs >25 µm for each DTC with any CPs >25 µm. Two-sided *t*-test. (D) Maximum length of CPs >25 µm for each DTC with any CPs >25 µm. Two-sided *t*-test. (E-I) Data for mid-L4 larvae. Representative images of DTCs in control (top) and DTC-specific *nmy-2* RNAi in mid-L4 larvae. (F) Bar plot showing the proportion of all DTCs with processes >25 µm. Black and gray bars represent DTCs with and without CPs >25 µm, respectively. Data pooled from three replicates after using Fisher's exact test to establish that pooling was appropriate. Fisher's exact test was used. (G) Number of CPs >25 µm in each DTC. Two-sided *t*-test. (H) Mean length of CPs >25 µm for each DTC with any CPs >25 µm. (I) Maximum length of CPs >25 µm for each DTC with any CPs >25 µm. In all panels where significance is indicated with asterisks: ****P<0.00001; ***P<0.0001; **P<0.001; ns, not significant (*P*≥0.01). Scale bars: 10 µm. In all panels, the DTC marker is *cpIs121(lag-2p::mNG::PH::F2A::rde-1).* In all dot plots, colors represent separate biological replicates; small dots represent a single DTC; large dots represent mean for that replicate. The total *n* for all replicates in each condition is indicated.

Non-muscle myosin activity positively correlates with membrane stiffness, whereby decreased concentration or de-activation of myosin decreases cortical tension and increases elasticity of actomyosin networks (Ebrahim et al., 2018; Ray et al., 2023; Vicente-Manzanares et al., 2009). To determine whether loss of *nmy-2* influences DTC elaboration, we performed DTC-specific RNAi against *nmy-2* from the L1 larval stage and observed that DTCs in *nmy-2* RNAi early adults had significantly more CPs >25 µm compared with controls (15 versus 11) (Fig. 7A,B) and that mean and maximum lengths of CPs >25 µm were longer in *nmy-2* RNAi than in controls (mean 45 versus 41; maximum 78 versus 60; Fig. 7A-D).

We reasoned that this early adult hyper-elaboration phenotype could result from either an increased rate or earlier onset of DTC elaboration, or both. The possibility of early DTC elaboration was suggested by findings of Agarwal et al. (2022, figure 1). Indeed, mid-L4 larvae fed DTC-specific *nmy-2* RNAi from the L1 stage showed precocious DTC elaboration (Fig. 7E). Specifically, 92% of *nmy-2* RNAi L4 DTCs had processes longer than 25 µm, compared with 33% of controls (Fig. 7F). Additionally, *nmy-2* RNAi DTCs showed a greater number and length of CPs >25 µm (three versus 0.4 average number, and 33 versus 29 µm and 42 versus 30 µm mean and maximum length, respectively) (Fig. 7G-I).

These observations suggest that non-muscle myosin is important for the timing of DTC elaboration.

### SAX-7/L1CAM and HMR-1 contribute to distinct aspects of DTC elaboration

We reasoned that any influence of germ cell flux on DTC elaboration would depend on DTC–germ cell adhesion. Based on well-characterized niche-stem cell adhesion candidates (Chen et al., 2013), we considered gap junctions, E-cadherin and L1CAM.

*C. elegans* innexins form gap junctions. However, our experiments with DTC-expressed innexins displayed phenotypic effects that hampered interpretation (see Fig. S2A,B).

**Fig. 8.**
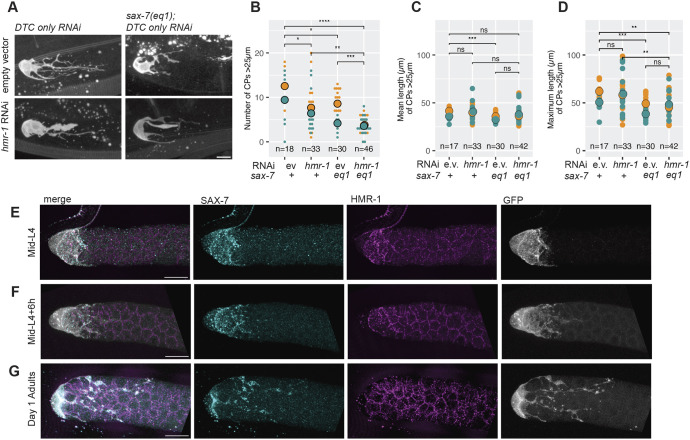
**Role of adhesive interactions in DTC elaboration.** (A) Representative images of L4+16 h DTCs with genotypes and RNAi conditions as indicated. Although not quantified, processes sometimes appeared thicker or smoother after DTC-specific RNAi of *hmr-1*, as shown in this image. Scale bar: 10 µm. (B) Number of CPs >25 µm in each DTC. Two-sided Wilcoxon test with Bonferroni. (C) Mean length of CPs >25 µm in each DTC with any CPs >25 µm. Two-sided *t*-test with Bonferroni correction. (D) Maximum length of CPs >25 µm in each DTC with any CPs >25 µm. Two-sided *t*-test with Bonferroni correction. (E-G) HMR-1 (magenta) and SAX-7 (cyan) staining in fixed gonads at mid-L4 (E), L4+6 h (F) and Day 1 adult (G). In all panels where significance is indicated with asterisks: ****P<0.00001; ***P<0.0001; **P<0.001; *P<0.01; ns, not significant (*P*≥0.01). In all panels, the DTC marker is *cpIs121(lag-2p::mNG::PH::F2A::rde-1(+)).* In all dot plots, colors represent separate biological replicates; small dots represent a single DTC; large dots represent mean for that replicate. The total *n* for all replicates in each condition is indicated. Scale bars: 10 µm.

Previously, it was shown that loss of *hmr-1/*E-cadherin and/or *sax-7/*L1CAM additively impact the formation of DTC short intercalating processes (Gordon et al., 2019). We examined the localization of HMR-1 and SAX-7 in the gonad at mid-L4, L4+6 h and in Day 1 Adults (see Materials and Methods). With the exception of L4, HMR-1 did not appear to be enriched at DTC–germ cell contacts. In contrast, SAX-7 appeared markedly enriched at DTC–germ cell contacts at each stage along the entire length of the DTC (Fig. 8E-G).

To determine the contribution of HMR-1 and SAX-7 to overall DTC elaboration, we quantified DTC process number and length (Gupta et al., 2024) in RNAi and/or mutant conditions. We observed that DTC-specific RNAi of *hmr-1* significantly reduced the number of CPs >25 µm (seven versus 11), but, for those processes >25 µm, the mean or maximum length were not affected, although the variability of length was elevated (Fig. 8). DTCs in *sax-7(eq1)* mutant worms displayed a similarly reduced number of CPs (seven versus 11), and they also displayed a reduced mean and maximum length of CPs (mean 35 versus 40 µm; maximum 45 versus 58 µm). Thus, whereas DTC-specific reduction of *hmr-1* influenced primarily the total number of processes, *sax-7* influenced both number and length. In accordance with this, we found that *hmr-1* RNAi in the *sax-7(eq1)* mutant additively reduced the average number of CPs from 11 in the control to 3.7, without an additional effect on length.

To ensure that the DTC-specific RNAi background {NK2115[*cpIs121(lag-2p::mNG::PH::F2A::rde-1) I; rrf-3(pk1426) II; rde-1(ne219) V*]} did not have independent effects on DTC elaboration, we examined DTC elaboration in a *sax-7(eq1)* mutant carrying a different DTC marker and without the additional genetic burden of mutants and transgenes that are required for DTC-specific RNAi (Linden et al., 2017; Qadota et al., 2007; Simmer et al., 2003). We observed fewer DTCs with CPs >25 µm in *sax-7(eq1)* worms (though the difference was not significant; *P*-value=0.094; Fig. S2C,D). However, the mean and maximum length of CPs >25 µm was significantly lower in *sax-7(eq1)* (mean: 33 versus 37 µm; maximum: 43 versus 49 µm) (Fig. S2E,F). Therefore, *sax-7(eq1)* influences DTC elaboration, although the phenotype is more severe in the genetic background that confers DTC-specific RNAi.

Importantly, the effects of these manipulations are relatively modest, suggesting that other adhesive interactions between the DTC and germ cells and/or the DTC and basement membrane promote DTC elaboration in conjunction with SAX-7 and HMR-1.

## DISCUSSION

We have shown that the dramatic morphological changes in the *C. elegans* hermaphrodite DTC occur within, and are subject to, the dynamic environment of niche-stem cell interaction and organ-level dynamics. Our time-course analysis demonstrates rapid DTC elaboration around the time of the L4-to-adult molt, but we do not find support for a heterochronic mechanism. Instead, our results demonstrate a strong correlation with the cessation of gonad elongation and overall germline flux. We demonstrate that DTC elaboration is affected by both bulk flux due to ovulation and more local germ cell flux due to distal germ cell proliferation. We further established that DTC-intrinsic membrane deformability and DTC–germ cell adhesion both play a role in DTC morphogenesis. We postulate that, provided sufficient DTC cortex flexibility and DTC adhesion to underlying germ cells, germline flux pulls the DTC cortex away from the cell body. DTC–germ cell adhesive interactions must be variable and dynamic as germ cells proliferate, consistent with variability in DTC shape. Our model does not rule out other contributions to DTC elaboration, such as active growth or signaling from germ cells.

In contrast to primarily active mechanisms, such as *C. elegans* anchor cell invasion (Hagedorn et al., 2014), our results suggest the contribution of a passive ‘hitch and tow’ mechanism more akin to retrograde extension (Cebul et al., 2020; Fan et al., 2019; Heiman and Bülow, 2024; Heiman and Shaham, 2009). In the classic neuronal example, amphid dendrite tips are anchored by DYF-7, and their cell bodies migrate away from the tips (Heiman and Shaham, 2009), whereas for the DTC the cell body remains stationary while the processes move away. A previous study postulated primarily active growth of DTC processes based on observation of spurts of DTC growth and the formation of rings along the length of adult processes (Wong et al., 2013). Although an active mechanism may contribute, the observed spurts could also result from underlying changes in the germ line, such as an ovulation event, and although the formation of rings of DTC membrane could reflect active adhesive dynamics between the DTC and individual germ cells, rings might also form during germ cell division.

Our analysis suggests a role for membrane stiffness in preventing premature process extension. Although overall germline flux is more dramatically ‘reversed’ at adult onset, some germ cells born in contact with the DTC during larval stages are displaced by proliferation or otherwise move proximally, even before the cessation of gonad elongation. Consistent with our model, we speculate that reduced *nmy-2* could facilitate premature DTC elaboration if sufficient adhesion exists between DTC membrane and underlying germ cells, such that any proximal germ cell movement could support DTC elaboration if the membrane is inappropriately pliable. Alternatively, reduced *nmy-2* could facilitate a more active aspect of DTC elaboration. Distinguishing these possibilities requires additional investigation.

Adhesion between the DTC and germ cells is an essential aspect of our model. We observed that HMR-1/E-cadherin and SAX-7/L1CAM each contribute to DTC elaboration. In contrast to the embryo, where SAX-7 and HMR-1 colocalize and work redundantly in blastomere compaction (Grana et al., 2010), SAX-7 and HMR-1 showed distinct enrichment patterns in the distal gonad. Our phenotypic analysis suggests that they may have differing roles as loss of *sax-7* affects both initiation and extension, whereas DTC-*hmr-1* RNAi affects process initiation. Alternatively, phenotypic differences may reflect differential sensitivity of the two aspects of DTC morphology to overall adhesion, or subtle effects of *sax-7* on the germ line. Additional adhesive mechanisms are likely involved in both initiation and extension of DTC processes. Furthermore, our observations from DTC *nmy-2* RNAi suggest that membrane stiffness is a limiting factor for DTC elaboration. The limit on DTC extensions could also be influenced by additional factors, such as sheath cell morphology (Tolkin et al., 2022).

One implication of our results is that further analysis of the mechanism by which the DTC elaborates – or similar scenarios in which cell shape is altered by contact with other cells – requires careful consideration of the effects of any manipulation on underlying germ cells. Manipulations, DTC-autonomous or otherwise, that interfere with germ cell development, proliferation, or bulk germline flux may be misinterpreted to be causal in DTC elaboration.

Another implication is general: proliferating cells that depend on direct cell–cell signaling may have a proliferative advantage if they retain contact with their signal-sending cell even as their numbers increase and undergo repositioning. DTC processes have been implicated in maintaining the proliferative pool by active Notch-receptor signaling in the adult germ line (Lee et al., 2016). By analogy, in niche-cancer stem cell scenarios (Ju et al., 2022; Wu et al., 2024) it is conceivable that proliferating tumor cells, by adhering to pliable niche cell membranes, could prolong growth-promoting signaling despite tumor growth.

## MATERIALS AND METHODS

### Worm strains and husbandry

*C. elegans* nematodes were grown on NGM solid media at 20°C (except where indicated) and fed *Escherichia coli* strain OP50, except for RNAi experiments, as described below. All strains analyzed are listed with full genotypes in Table S1. Strains generated for this publication are GC1652, GC1628, GC1637, GC1701, GC1718, GC1724, GC1738, GC1774, GC1805 and GC1811. All strains were based on Bristol N2 (RRID:WB-STRAIN:WBStrain00000001). Many experiments required access to information on WormBase (Davis et al., 2022).

### Imaging

For live imaging, 15-20 worms were placed onto a 3% agarose pad on a glass slide and anesthetized using 15 mM levamisole in M9. For fixed, DAPI-stained worms, we mounted 5-10 µl of worms in suspension in Vectashield with DAPI (H-1000, Vector Laboratories) onto a 3% agarose pad on a glass slide. We used a Nikon W1 spinning disk confocal microscope with 60× oil immersion magnification. For live images, we used a step size of 0.3 µm. For DAPI-stained images, we used a step size of 1 µm.

### Sholl analysis

Sholl analysis quantifies the complexity of a cell's shape by defining a center point ringed by concentric radii spaced at defined intervals and counting the number of times the circumference at each radial distance from the center point intersects the cell. We performed Sholl analysis using the SNT package in ImageJ (Ferreira et al., 2014). We spaced radii 1 µm apart with no averaging proximal or distal to each radius. We set the center point manually at the distalmost end of the gonad and measured out to a final distance of 100 µm for all samples.

### Manual marking of continuous processes

We measured all continuous processes ending at least 25 µm away from the distal end of the DTC, according to the methods described by Gupta et al. (2024).

### Time-course analyses

For each time-course analysis, we collected triplicate cohorts of worms at the mid-L4 stage (L4.5 based on Mok et al., 2015) and sampled from the cohorts to image DTC and/or gonads in 10-20 worms per replicate at the time points indicated. Exact sample numbers are included in the figures.

### RNAi by bacterial feeding

RNAi experiments were conducted as described by Timmons et al. (2001). Briefly, *E. coli* strain HT115 carrying either an empty vector or a vector containing a sequence of the gene of interest were streaked out onto solid LB plates supplemented with 100 µg/ml ampicillin and 10 µg/ml tetracycline and grown overnight at 37°C. Single colonies were used to inoculate liquid LB cultures supplemented with 100 µg/ml ampicillin and grown at 37°C with shaking for 12-16 h. This liquid culture was used to seed NGM plates supplemented either with IPTG to a final concentration of 1 µM (for knockdown of *lin-41*, and associated controls) or with 0.5% β-lactose (for all other experiments). Seeded RNAi plates were kept at room temperature for 24 h prior to adding worms.

We obtained large populations of synchronized L1 larvae by allowing eggs obtained by hypochlorite treatment of gravid adults to hatch in M9 overnight on a shaker at 20°C. L1 larvae were transferred to RNAi plates seeded with *E. coli* strain HT115 carrying either the empty vector (L4440 or T4440 plasmids as appropriate) as the negative control, a plasmid targeting *skn-1* or *bli-3* for RNAi as the positive control, or a plasmid carrying the sequence of the gene of interest, as indicated in the text and figure legends. Complete penetrance of the *skn-1* maternal lethal Emb phenotype or of the *bli-3* Bli phenotype confirmed the efficacy of RNAi reagents in each experimental replicate. For the experiments knocking down *lin-41*, we further confirmed the efficacy of RNAi by measuring the penetrance and expressivity of precocious heterochronic phenotypes in CB4088 *him-5(e1490)* adult males. The penetrance of the *lin-41* reduction-of-function over-retraction ORE phenotype was assessed using the protocol described by Burnett et al. (2023).

Knockdown of *lin-41* was performed using clone X-5B19 obtained from the Ahringer library (Kamath et al., 2003). Knockdown of *nmy-2* was performed using pGC748, a plasmid containing the *nmy-2* coding region between two T7 RNA polymerase sites in the T444T vector (Sturm et al., 2018). We amplified *nmy-2* from N2 genomic DNA using primers that included tailing sequences for Gibson assembly (Agarwal et al., 2022) and inserted into a linearized T444T vector (Sturm et al., 2018) using Gibson assembly (Gibson et al., 2009). Primer sequences are given in Table S1.

### Measuring gonad elongation

We used Nomarski optics at 40× magnification to obtain images of distal gonads and the vulva in the same frame. We used the center of the vulva as a landmark, the vulval midline, to place a line perpendicular to the body axis of the worm, and then measured the distance at a right angle from the distal end of one gonad arm to the vulval midline. If the distal end of the gonad was on the same side of the vulval midline as the proximal end of that same gonad, the number we reported was negative; if the distal end of the gonad had crossed the line, the number reported was positive. All measurements are in microns. For experiments involving mutants in which gonad elongation was disrupted (*mig-39* and *hlh-12*), we excluded gonads that had gross confounding pathfinding defects or that had not completed the U turn. In all cases, mutant and control worms were grown and assayed in parallel.

### Counting nuclei of the proliferative zone and assessing arrest of PZ nuclei

To collect images of DAPI-stained gonads, we used a Nikon W1 confocal microscope, with 40× magnification and a step size of 1 µm. We counted PZ nuclei manually in ImageJ using the built-in multi-point tool. In experiments using *emb-30* mutants at the restrictive temperature, we counted all nuclei in the PZ and then performed a separate count only of those nuclei in metaphase (recognizable by the characteristic compacted appearance) to obtain the proportion of nuclei in metaphase for each gonad arm. In experiments using *glp-4* mutants at the restrictive temperature, we counted all nuclei and then performed a separate count only of those nuclei in prometaphase (recognizable by the characteristic dotted nuclear appearance) to obtain the proportion of nuclei in prometaphase for each gonad arm.

### Use of low temperature for germline arrest

Prior reports of using cold temperature to arrest cell divisions in *C. elegans* used a temperature of 6°C (Sulston and Horvitz, 1977). We performed our experiments at 10°C, the minimum stable temperature we could reach. Worms were grown at 20°C until L4+6 h. NGM plates seeded with OP50 were pre-cooled for at least 2 h at 10°C and staged worms were transferred to the cooled plates and then placed at 10°C.

### Immunohistochemistry

Staging of mid-L4 and L4+6 h worms was carried out as described above. Gravid adult worms were picked from mixed-stage plates where all adults were <36 h post-mid-L4. These were considered ‘Day 1 Adults’. Gonads were extruded in M9 with 1 mM levamisole and fixed at room temperature for 10 min in 10% paraformaldehyde with 1 mM EGTA, 5 mM PIPES, 2.5 mM HEPES and 2 mM MgCl_2_ in PBS with 0.1% Tween 20, then transferred to ice-cold methanol for 10 min. Staining was carried out as previously reported (Tolkin et al., 2022). Antibodies used were: rabbit anti-HMR-1 (1:10,000; Klompstra et al., 2015), mouse anti-SAX-7 (1:15; 2.5 µg/ml; Developmental Studies Hybridoma Bank; Klompstra et al., 2015). Secondary antibodies used were: goat-anti-rabbit Alexa Fluor 555 (1:100; abcam, ab150078), goat-anti-mouse Alexa Fluor 647 (1:100; abcam, ab150115). DTCs were visualized by the transgenic GFP fluorescence that persisted through fixation and staining. During our experiments, we noticed that the GFP from *cpIs121* (Linden et al., 2017) persisted better through fixation than *qIs57*; therefore, the staining data shown here is in worms carrying *cpIs121* as a DTC marker.

### Quantification and statistical analysis

To determine significance between pair-wise conditions for the proportion of DTCs with CPs >25 µm, we used Fisher's exact test; to determine significance between conditions for the number of CPs >25 µm for each DTC, we used a two-sided Wilcoxon test; to determine significance between conditions for mean length of CPs >25 µm and maximum length of CPs >25 µm, we used a two-tailed *t*-test. When reporting the mean and maximum CP length, we did not include DTCs that had no CPs >25 µm. All sample numbers (*n*) are reported on each graph.

## Supplementary Material

10.1242/develop.203019_sup1 Supplementary information
