# Supplementary material for: A role for organ level dynamics in morphogenesis of the C. elegans hermaphrodite distal tip cell
Source: Development. 2024 Oct 9;151(19):dev203019. doi: 10.1242/dev.203019 (PMC11488634; doi:10.1242/dev.203019)
Supplement: Supplementary information [file develop-151-203019-s1.pdf]

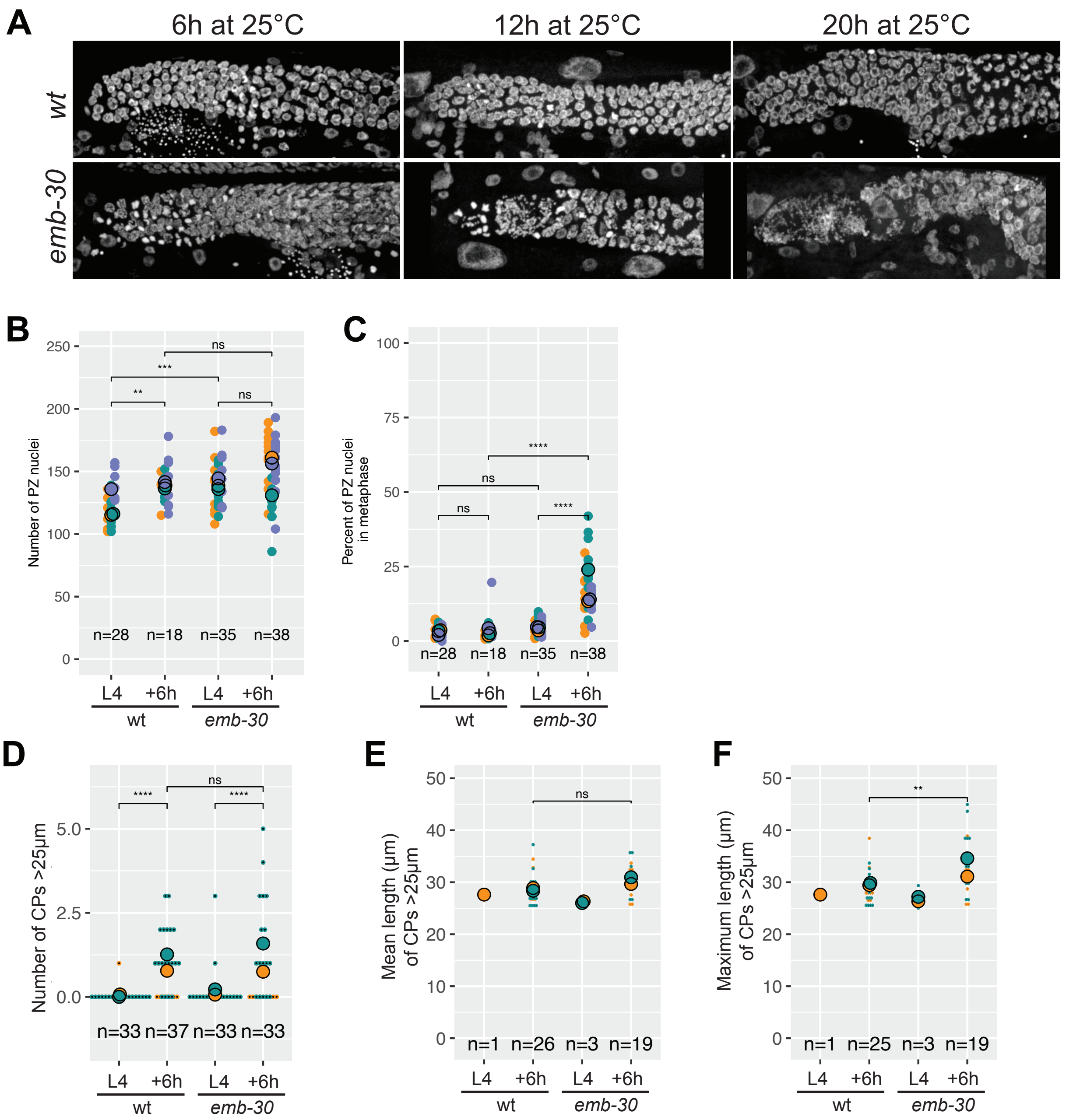

Fig. S1. *emb-30(tn377)* mutation leads to severe nuclear abnormality in the distal gonad when kept at the restrictive temperature for long periods of time.

A) Representative images of DAPI-stained gonads in wild-type and *emb-30(tn377)* worms that were grown at 20°C and then shifted at the mid-L4 larval stage to 25°C for 6, 12 or 20 hours, as indicated.

B) Superplot showing the number of PZ nuclei in DAPI-stained gonads. Two-sided T-test was used.

C) The percent of PZ nuclei in prometaphase. Two-sided T-test was used.

D) The number of CPs >25 µm in each DTC for the genotypes and time-points indicated. Two-sided Wilcoxon test was used.

E) The mean length of CPs >25 µm in each DTC that showed any CPs >25 µm. Two-sided T-test was used.

F) The maximum length of CPs >25 µm in each DTC that showed any CPs >25 µm. Two-sided T-test was used.

Scale bars 10µm. In all panels, the DTC marker is *qIs57(lag-2p::GFP).* In all dot-plots, colors represent separate biological replicates; each small dot represents a single DTC; the large dots represent that mean for that replicate. The total n for all replicates in each condition is shown on the graphs.

Development • Supplementary information

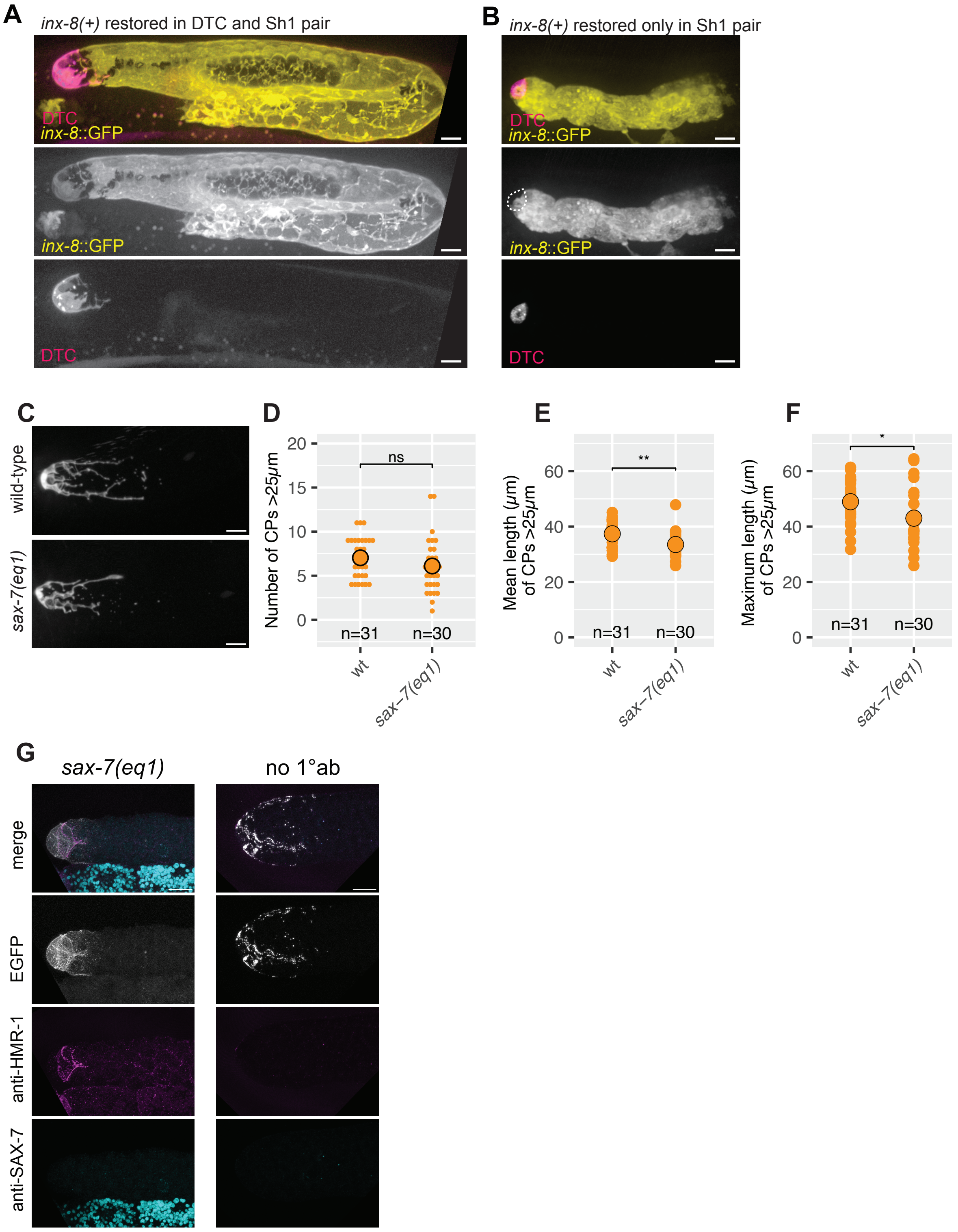

A
inx-8(+) restored in DTC and Sh1 pair
DTC
inx-8::GFP
inx-8::GFP
DTC
B
inx-8(+) restored only in Sh1 pair
DTC
inx-8::GFP
inx-8::GFP
DTC
C
wild-type
sax-7(eq1)
D
Number of CPs >25μm
ns
n=31
n=30
wt
sax-7(eq1)
E
Mean length (μm) of CPs >25μm
**
n=31
n=30
wt
sax-7(eq1)
F
Maximum length (μm) of CPs >25μm
*
n=31
n=30
wt
sax-7(eq1)
G
sax-7(eq1)
no 1°ab
merge
EGFP
anti-HMR-1
anti-SAX-7

Fig. S2. Investigation of candidate adhesion molecules.

(A-B) Top panels: merge; middle panels: *lag-2p::inx-8::GFP* expression in the DTC and *lim-7p::inx-8::GFP* expression in Sh1. Bottom panels: *lag-2p::mCherry* DTC marker (*naIs37*).

Panels A and B: Innexin experiments.
The DTC expresses two gap junction proteins, innexins INX-8 and INX-9 which act redundantly to promote progenitor accumulation and to direct somatic gonad architecture (Starich et al., 2014; Starich and Greenstein, 2020; Tolkin et al., 2022). Using transgenes expressing INX-8::GFP under the control of DTC- (*lag-2p*) or sheath- (*lim-7p*) promoters in the *inx-8 inx-9* double mutant, we compared DTC morphology in worms carrying both arrays vs. worms carrying only the sheath expression array. Unfortunately, abnormal gonad architecture and/or reduced germline proliferation precluded interpretation of these experiments.

A) Representative image of a distal gonad in a *inx-8(0) inx-9(0)* double mutant worm bearing two extrachromosomal arrays, one restoring *inx-8(+)::GFP* in the DTC and one in most of the gonadal sheath, including Sh1 pair.
B) Representative image of a distal gonad in a *inx-8(0) inx-9(0)* double mutant bearing an with extrachromosomal array restoring *inx-8(+)::GFP* only in the sheath (including Sh1 pair), but not in the DTC.

Panels C-F: Loss of *sax-7* in a different genetic background.

C) Representative images of L4p24h DTCs carrying the *qIs57(lag-2p::GFP)* DTC marker and either *sax-7(+)* or *sax-7(eq1)*.
D) Superplot showing the number of CPs >25 µm in each DTC. Two-sided Wilcoxon test was used.
E) Superplot showing the mean length of CPs >25 µm in each DTC that showed any CPs >25 µm. Two-sided T-test was used.
F) Superplot showing the maximum length of CPs >25 µm in each DTC that showed any CPs >25 µm. Two-sided T-test was used.

Panel G-H: Antibody staining controls.

G) Control for anti-SAX-7 antibody. Representative image of an extruded and stained L4 gonad of a *sax-7(eq1)* mutant. While anti-HMR-1 staining pattern is clear, there is no anti-SAX-7 staining.
H) No primary antibody staining controls. No primary antibody was used, only secondary antibody. No fluorescent signal is visible from either anti-rabbit Alexa555 or anti-mouse Alexa647.

In all panels where significance is indicated with asterisks: **** p-value < 0.00001; *** p-value < 0.0001; ** p-value < 0.001; * p-value < 0.01; 'ns' p-value ≥0.01. Scale bars are 10µm. In all dot-plots, colors represent separate biological replicates; each small dot represents a single DTC; the large dots represent that mean for that replicate. The total n for all replicates in each condition is shown on the graphs.

Development • Supplementary information

Table S1. Reagents and Strains Used

REAGENT or RESOURCE	SOURCE	IDENTIFIER
Chemicals, peptides, and recombinant proteins		
IPTG (Isopropyl β-D-1-thiogalactopyranoside, IPTG, Isopropyl β-D-thiogalactoside)	Sigma Aldrich	Cat#I6758
β-lactose (β-D-Gal-(1→4)-β-D-Glc)	Sigma Aldrich	Cat#L3750
Experimental models: Organisms/strains		
qIs57(lag-2p::GFP[pJK590]) II	Byrd et al.(Byrd et al., 2014)	JK2869
lin-41(bx37) I; qIs57(lag-2p::GFP[pJK590]) II; him-5 (e1490) V	This work	GC1652
qIs57 (lag-2::GFP[pJK590]) II; mig-39(tk107) III	This work	GC1718
qIs57(lag-2::GFP[pJK590]) II; hlh-12/mig-24(tk68)	This work	GC1724
acy-4(ok1806) V; naIs37(lag-2p::mCherry); tnEx42[acy-4::GFP + rol-6(su1006)]	Govindan et al.(Govindan et al., 2009)	DG5310
naIs37(pGC457[lag-2p::mCherryPH unc-119(+)]); unc-119(ed3)	Pekar et al.(Pekar et al., 2017)	GC1038
gld-2(q497) gld-1(q485)/hT2gfp I; qIs57 II; +/hT2gfp III	This work	GC1628
qIs57 (lag-2p::GFP[pJK590]) II; emb-30 (tn377) III	This work	GC1637
glp-4(bn2) II; qIs57 (lag-2p::GFP [pJK590]) II	This work	GC1701
qIs57 (lag-2p::GFP[pJK590]) II; his-72(his2[HIS-72::Dendra2]) III	This work	GC1738
cpIs121[lag-2p::mNG::PH::F2A::rde-1] I; rrf-3(pk1426) II; rde-1(ne219) V	Linden et al. (Linden et al., 2017)	NK2115
naIs37 (pGC457[lag-2p::mCherryPH unc-119(+)]) I; inx-8(tn1474) inx-9(ok1502) IV; tnEx201[myo-2p::TdTomato; lag-2p::inx-8::gfp; N2 DNA]; tnEx203[lim-7p::inx-8::gfp; str-1::gfp]	This work	GC1774
qIs57(lag-2p::GFP) II; sax-7(eq1) IV	This work	GC1805
cpIs121 (lag-2p::mNG::PH::F2A::rde-1) I; rrf-3(pk1426) II; sax-7(eq1) III; rde-1(ne219) V	This work	GC1811
Oligonucleotides		
Forward primer for nmy-2 with T444T overhangs TCTGATATCATCGATGAA TTCGAGCTCCACTCAGGCTGTTCTG CTCAATG	Agarwal et al. (Agarwal et al., 2022)	
Reverse primer for nmy-2 with T444T overhangs CCCTCGAGGTCGACGGT ATCGATAAGCTTGGAGCGAATCTCT GGAACGAC	Agarwal et al.(Agarwal et al., 2022)	
Forward primer for vector with overhangs of nmy-2 TCCGAAGTACGTCGTTCCA GAGATTCGCTCCAAGCTTATCGATA CCGTCGAC	Agarwal et al.(Agarwal et al., 2022)	
Reverse primer for vector with nmy-2 overhangs GCAACAGCGTCATTGAG CAGAACAGCCTGAGTGGAGCTCGA ATTCATCGATG	Agarwal et al.(Agarwal et al., 2022)	
Recombinant DNA		
RNAi vector against *nmy-2* in T444T	This work	pGC768
RNAi vector against *hmr-1* in L4440	Kamath et al.(Kamath et al., 2003)	X-5B19

Development • Supplementary information

Supplementary References

Agarwal, P., Shemesh, T., Zaidel-Bar, R., 2022. Directed cell invasion and asymmetric adhesion drive tissue elongation and turning in C. elegans gonad morphogenesis. Dev Cell. https://doi.org/10.1016/j.devcel.2022.08.003

Byrd, D.T., Knobel, K., Affeldt, K., Crittenden, S.L., Kimble, J., 2014. A DTC Niche Plexus Surrounds the Germline Stem Cell Pool in Caenorhabditis elegans. PLoS One 9, e88372. https://doi.org/10.1371/journal.pone.0088372

Govindan, J.A., Nadarajan, S., Kim, S., Starich, T.A., Greenstein, D., 2009. Somatic cAMP signaling regulates MSP-dependent oocyte growth and meiotic maturation in C. elegans. Development 136, 2211–2221. https://doi.org/10.1242/dev.034595

Kamath, R.S., Fraser, A.G., Dong, Y., Poulin, G., Durbin, R., Gotta, M., Kanapin, A., Le Bot, N., Moreno, S., Sohrmann, M., Welchman, D.P., Zipperlen, P., Ahringer, J., 2003. Systematic functional analysis of the Caenorhabditis elegans genome using RNAi. Nature 421, 231–237. https://doi.org/10.1038/nature01278

Linden, L.M., Gordon, K.L., Pani, A.M., Payne, S.G., Garde, A., Burkholder, D., Chi, Q., Goldstein, B., Sherwood, D.R., 2017. Identification of regulators of germ stem cell enwrapment by its niche in C. elegans. Dev Biol 429, 271–284. https://doi.org/10.1016/j.ydbio.2017.06.019

Pekar, O., Ow, M.C., Hui, K.Y., Noyes, M.B., Hall, S.E., Hubbard, E.J.A., 2017. Linking the environment, DAF-7/TGFβ signaling and LAG-2/DSL ligand expression in the germline stem cell niche. Development 144, 2896–2906. https://doi.org/10.1242/dev.147660

Starich, T.A., Greenstein, D., 2020. A limited and diverse set of suppressor mutations restore function to inx-8 mutant hemichannels in the caenorhabditis elegans somatic gonad. Biomolecules 10, 1–15. https://doi.org/10.3390/biom10121655

Starich, T.A., Hall, D.H., Greenstein, D., 2014. Two classes of gap junction channels mediate soma-germline interactions essential for germline proliferation and gametogenesis in caenorhabditis elegans. Genetics 198, 1127–1153. https://doi.org/10.1534/genetics.114.168815

Tolkin, T., Mohammad, A., Starich, T.A., Nguyen, K.C., Hall, D.H., Schedl, T., Jane Albert Hubbard, E., Greenstein, D., 2022. Innexin function dictates the spatial relationship between distal somatic cells in the Caenorhabditis elegans gonad without impacting the germline stem cell pool 11, 74955. https://doi.org/10.7554/eLife
